# Non-invasive Motor Cortex Neuromodulation Reduces Secondary Hyperalgesia and Enhances Activation of the Descending Pain Modulatory Network

**DOI:** 10.3389/fnins.2019.00467

**Published:** 2019-05-08

**Authors:** Timothy J. Meeker, Michael L. Keaser, Shariq A. Khan, Rao P. Gullapalli, David A. Seminowicz, Joel D. Greenspan

**Affiliations:** ^1^Department of Neurosurgery, Johns Hopkins Medicine, Baltimore, MD, United States; ^2^Department of Neural and Pain Sciences, School of Dentistry, University of Maryland, Baltimore, Baltimore, MD, United States; ^3^Program in Neuroscience, University of Maryland, Baltimore, Baltimore, MD, United States; ^4^Center to Advance Chronic Pain Research, University of Maryland, Baltimore, Baltimore, MD, United States; ^5^Department of Diagnostic Radiology and Nuclear Medicine, University of Maryland, Baltimore, Baltimore, MD, United States

**Keywords:** motor cortex neuromodulation, pain model, secondary hyperalgesia, BOLD fMRI, event-related fMRI, human, pain, transcranial direct current stimulation

## Abstract

Central sensitization is a driving mechanism in many chronic pain patients, and manifests as hyperalgesia and allodynia beyond any apparent injury. Recent studies have demonstrated analgesic effects of motor cortex (M1) stimulation in several chronic pain disorders, yet its neural mechanisms remain uncertain. We evaluated whether anodal M1 transcranial direct current stimulation (tDCS) would mitigate central sensitization as measured by indices of secondary hyperalgesia. We used a capsaicin-heat pain model to elicit secondary mechanical hyperalgesia in 27 healthy subjects. In an assessor and subject-blind randomized, sham-controlled, crossover trial, anodal M1 tDCS decreased the intensity of pinprick hyperalgesia more than cathodal or sham tDCS. To elucidate the mechanism driving analgesia, subjects underwent fMRI of painful mechanical stimuli prior to and following induction of the pain model, after receiving M1 tDCS. We hypothesized that anodal M1 tDCS would enhance engagement of a descending pain modulatory (DPM) network in response to mechanical stimuli. Anodal tDCS normalized the effects of central sensitization on neurophysiological responses to mechanical pain in the medial prefrontal cortex, pregenual anterior cingulate cortex, and periaqueductal gray, important regions in the DPM network. Taken together, these results provide support for the hypothesis that anodal M1-tDCS reduces central sensitization-induced hyperalgesia through the DPM network in humans.

## Introduction

Moderate to severe chronic pain afflicts 17–33% of adults in western countries (Bouhassira et al., 2008; Von Korff et al., 2016). Treatments are infrequently effective for chronic pain (Attal and Bouhassira, 2015). Studies have found motor cortex (M1) stimulation to ameliorate chronic pain. Pain mitigating neuromodulatory methods include epidural motor cortex stimulation (EMCS), repetitive transcranial magnetic stimulation (rTMS), and transcranial direct current stimulation (tDCS) (Tsubokawa et al., 1993; Lefaucheur et al., 2001; Fregni et al., 2006). Most successful trials of M1 neuromodulation have involved neuropathic pain patients (Fontaine et al., 2009; O’Connell et al., 2014). Importantly, all successful M1 tDCS trials in chronic pain patients have favored anodal tDCS or high frequency rTMS, both methods that favor increasing excitability of the cortex, while cathodal tDCS has only occasionally demonstrated analgesic effects in assays of phasic acute pain (Mylius et al., 2012; Vaseghi et al., 2014, 2015). This led us to evaluate the efficacy of M1 tDCS on symptoms evoked by the capsaicin-heat pain (C-HP) model (Anderson et al., 2002; Furman et al., 2018). We expected initial effects of pain alleviation after one 20 min session, since motor cortex excitability, measured by motor-evoked potentials, is altered after 5 min of M1 tDCS (Nitsche and Paulus, 2000).

The C-HP model is driven by sustained peripheral nociceptive input in healthy volunteers (Anderson et al., 2002; Lötsch et al., 2015). Enhanced responses of spinal dorsal horn neurons, a form of central sensitization, to mechanical stimuli after capsaicin exposure mediates secondary hyperalgesia in the model (LaMotte et al., 1991; Simone et al., 1991). Central sensitization maintained by peripheral nociceptive input is a predominant mechanism of pain in neuropathic pain patients (Gracely et al., 1992; Haroutounian et al., 2014). A recent meta-analysis found capsaicin models outperform all other pain models in predicting clinical efficacy of novel and established therapeutics (Lötsch et al., 2014). Capsaicin sensitization mimics the pattern of sensory symptoms in neuropathic pain (Lötsch et al., 2015). This model’s success in analgesic evaluation coupled with the ability to assay psychophysical outcomes of central and peripheral sensitization makes this an ideal test of anodal M1 tDCS.

Animal studies of EMCS demonstrated anti-nociception is accompanied by alterations in neural activity in the spinal dorsal horn, periaqueductal gray (PAG), and prefrontal cortices (Tsubokawa et al., 1991; Pagano et al., 2011, 2012; Kudo et al., 2014). Pain reduction by M1 rTMS correlates with and predicts the clinical success of EMCS, suggesting these interventions have similar mechanisms (Hosomi et al., 2008; André-Obadia et al., 2014). Studies in chronic pain patients have implicated increased activity in the ACC and medial prefrontal cortex (MPFC), anterior midcingulate cortex (aMCC), and PAG, regions of the descending pain modulatory (DPM) network, in the mechanism of EMCS (García-Larrea et al., 1999; Peyron et al., 2007). Together, these findings implicate a model whereby enhancing neural activity in motor cortex by tDCS activates neurons in the aMCC and ACC leading to the descending inhibition of pain.

The current study in healthy volunteers is the first to show an analgesic effect on centrally mediated hyperalgesia by anodal tDCS and couples this to activation of DPM network. We predicted anodal tDCS would reduce perceptual correlates of central sensitization to a greater extent than cathodal or sham tDCS. Specifically, our primary outcome was intensity of secondary mechanical hyperalgesia developed after application of anodal, cathodal or sham M1 tDCS. For the neuroimaging phase, we specifically hypothesized anodal tDCS would be associated with enhanced activation of the DPM network to painful stimuli. We tested this hypothesis by measuring the evoked BOLD response to hyperalgesic stimuli using regions of interest (ROIs) in the pACC, MPFC, aMCC, and PAG. Secondarily, we assessed an ROI in the somatosensory cortex contralateral to the mechanical stimuli.

## Materials and Methods

An initial screening session was conducted, since not all subjects develop heat allodynia and mechanical hyperalgesia to the C-HP model (Liu et al., 1998). Following confirmation of eligibility, three experimental sessions were conducted where transcranial direct current stimulation (tDCS) was applied targeting the motor cortex. We applied anodal, cathodal and sham tDCS in an assessor- and subject-blinded crossover manner in a randomized order. Before the study began on March 20, 2012, the lead author generated a simple serial randomization for the first 24 subjects to be applied as soon as subjects passed the screening session level of the study. The same process was applied separately for the first 15 randomizations of the sequential MRI sessions. A study technician, who applied the tDCS and performed some sensory testing during the screening sessions, determined the within subject tDCS condition randomization and remained unblinded throughout the study. This technician kept the randomization orders and the lead author performed all assessments after intervention application and was ignorant of the randomization. During the study, additional randomizations were generated June 29, 2013 for ten additional potential subjects by the unblinded study technician. During each experimental session we applied the C-HP model and assessed pain intensity (thermal allodynia) during tDCS, and measured extent and intensity of pinprick hyperalgesia and residual pain up to 65 min after tDCS. fMRI was conducted before and after inducing sensitization using the C-HP model in a subset of 15 subjects during anodal, cathodal or sham tDCS. All subjects provided written informed consent, and all procedures were approved by the University of Maryland, Baltimore Institutional Review Board for the Protection of Human Subjects.

### Subject Characteristics

Prior to conducting the study (in 2012), we conducted a power analysis using data from the most similar capsaicin motor cortex stimulation study that measured pain intensity ratings after exposure to capsaicin available (Tamura et al., 2004). This study presented many differences compared to our planned study including method of capsaicin application, primary outcome of thermal allodynia, and mode of motor cortex stimulation (rTMS), however, we determined that we would need data from 25 subjects to achieve a similar effect size as the Tamura et al. (2004) study with a power of 0.8 and an alpha of 0.95.

We screened 50 healthy subjects (27 females) to attain 33 subjects that developed static mechanical hyperalgesia and met all eligibility criteria (Supplementary Table S1). Subjects were enrolled from April 10, 2012 to August 14, 2013. After the conclusion of the last randomized sensory session on (January 24, 2014) the study team was unblinded to the sensory session data only. The final MRI session was May 7, 2014, after which the study team was unblinded to the MRI data. During the study there were no changes made to the protocol except that subjects who tested positive for marijuana use were allowed to continue enrollment, when they were able to test negative for illicit drugs at a subsequent session. Three subjects who only provided data from one intervention session and three subjects that reported a history of disorders which were excluded from the study were not included in the data analysis. This provided a final dataset of 27 subjects who experienced at least 2 intervention sessions.

We ensured continuing eligibility with a random urine drug screen which detected tetrahydrocannabinol (THC), cocaine, methamphetamine, amphetamine, ecstasy, heroin, phencyclidine, benzodiazepines, methadone, barbiturates, tricyclic antidepressants, or oxycodone. We allowed subjects who tested positive for THC to be retested 4 weeks later. They were allowed back into the study if they no longer tested positive for THC. In these cases, we tested the subjects before every session. This occurred in four male subjects, two of which were re-enrolled after producing negative urine drug screens. This was tolerated since 36% of college-aged adults in the United States report annual marijuana use (Johnston et al., 2014).

**FIGURE 1:**
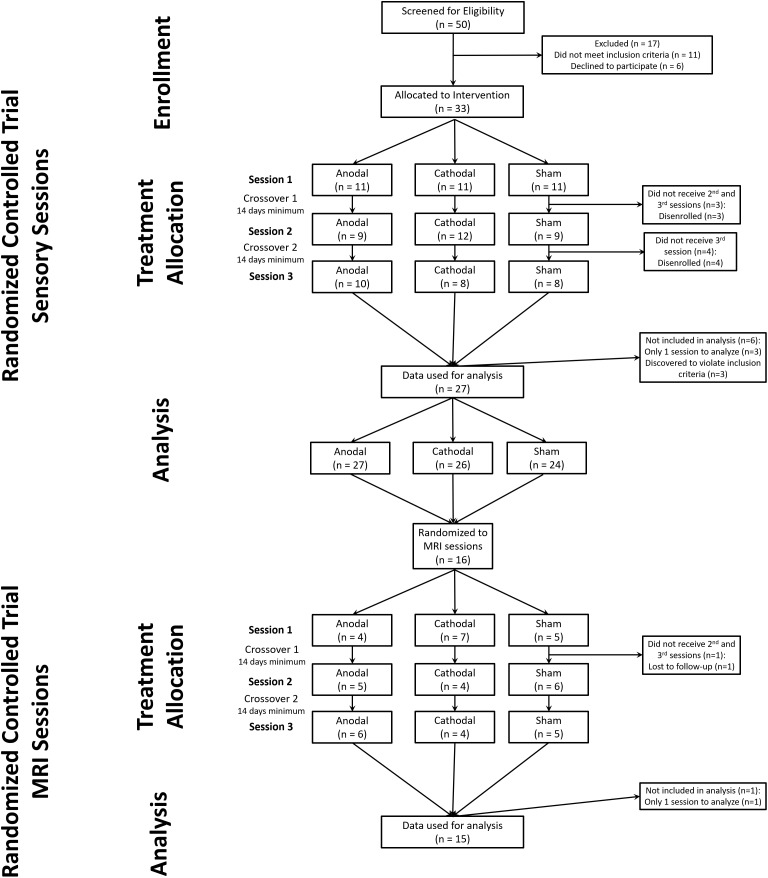
CONSORT diagram for study. We screened 50 potential subjects of which 33 were allocated to a randomized intervention. During the study 6 subjects failed to maintain eligibility or were lost to follow-up. After one MRI session with sham tDCS one subject was lost to follow-up.

We obtained complete data sets from 24 subjects (11 females) (CONSORT diagram: Figure 1) (Boutron et al., 2017). We included data from an additional 3 subjects (males) who completed 2 of 3 experimental sessions (overall group: age range 20–35, mean 25).

### Screening Session

We measured warmth detection thresholds (WDTs) and heat pain thresholds (HPTs) with a commercially available stimulator (Pathway; Medoc; Ramat Yishai, Israel) using the method of limits (Greenspan, 2013). A 3 by 3 cm contact area stimulator was placed on the subject’s lower left foreleg at 32°C and increased at a rate of 0.5°C/s until the subject pushed a mouse button indicating they “felt a change in temperature” (WDT) or when warmth “becomes painful” (HPT). This procedure was repeated four times for both WDTs and HPTs. We measured Mechanical pain thresholds (MPTs) using standardized weighted probes (16, 32, 64, 128, 256, and 512 mN of force) and the Chaplan-Dixon method for 50% threshold determination, which is an optimized variant on the up-down staircase method (Chaplan et al., 1994). We evaluated suprathreshold mechanical pain ratings by having subjects rate pain intensity to four different weighted mechanical probes (64, 128, 256, and 512 mN of force), which we applied in a counterbalanced order. Subjects rated their pain intensity on a numerical rating scale (NRS) with verbal anchors and numbers ranging from 0 to 100 by 10’s (Greenspan et al., 2003).

Sustained pain was induced by applying 1 g of 10% capsaicin cream within a 2.5 by 2.5 cm window cut into a Tegaderm^TM^ bandage to the anterior of the lower left leg. A second Tegaderm^TM^ bandage was applied over top to contain the cream (C-HP model) (Anderson et al., 2002; Furman et al., 2018). A thermode was placed over the area and maintained a temperature of 32°C. After a 15-min exposure we ramped the thermode to a predetermined target temperature for 23 min. The target temperature was between the subject’s WDT and HPT. This procedure does not cause tissue damage (Moritz and Henriques, 1947).

**FIGURE 2:**
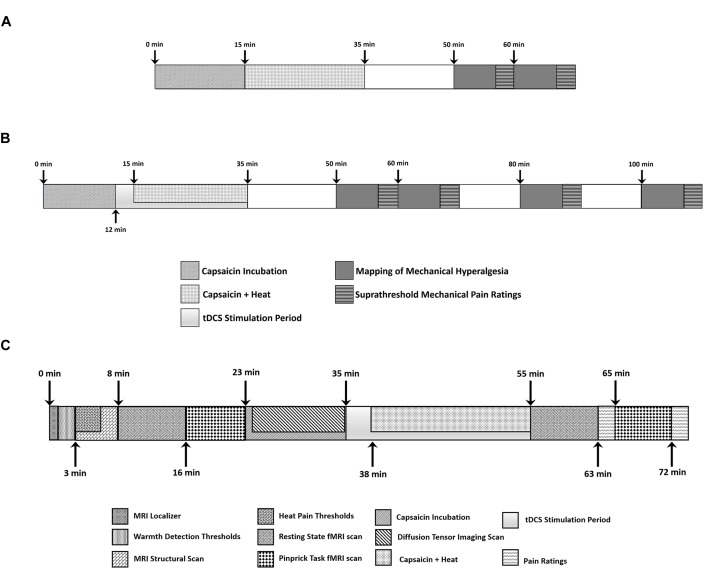
Timeline of events during **(A)** screening session and **(B)** each experimental session where subjects experienced anodal, cathodal or sham tDCS. **(C)** Each subject underwent three MRI scanning sessions where they experienced anodal, cathodal or sham tDCS.

During the C-HP exposure, subjects rated their pain intensity every minute for 35 min. Subjects additionally rated their pain intensity in absence of stimulation at 15 and 25 min after capsaicin removal. We measured the area of secondary hyperalgesia with a 128 mN weighted probe by probing from outside the hyperalgesic zone toward capsaicin exposure site, along eight radial axes, in 1 cm increments at 15 and 25 min after capsaicin removal. When the subject reported pain at two consecutive radial points, we marked the first point. If a subject did not report pain at an individual axis, we tested that axis again. If after the second probing no pain was reported, we marked the edge of the capsaicin exposure site. Markings were transposed to an acetate sheet for digitizing and measurement via ImageJ to count the pixels within the area of hyperalgesia, which were then divided by the number of pixels contained within 1 cm^2^. We subtracted from this area the capsaicin exposure area. This resulted in the area of secondary hyperalgesia. Additionally, at 15 and 25 min after capsaicin removal, we probed 1 cm outside of the capsaicin exposure site with calibrated weighted probes (64, 128, 256, and 512 mN of force) and had subjects rate pain intensity (Screening session overview: Figure 2A).

### Experimental Sessions

During the next three experimental sessions, we followed a protocol similar to the screening session. An unblinded study team member programmed the tDCS to apply anodal, cathodal or sham tDCS in a randomized crossover design. We imposed a minimum interval of 2 weeks between each session to mitigate carry-over effects of tDCS or the C-HP model.

At the beginning of each session, we assessed the subject’s safety by having her or him fill out a tDCS safety questionnaire. The questionnaire assessed if the subjects ever had the following: experienced tDCS, adverse reaction to tDCS, seizure, unexplained loss of consciousness, stroke, serious head injury, surgery to their head, brain-related neurological illness, illness that may have caused brain damage, brain injury or frequent or severe headaches. The questionnaire also determined if the subjects had any metal in their head, not including dental fillings or hardware, any implanted medical devices, were taking any medications, may be pregnant, or if anyone in their family had seizures. During the first session, the research technician measured the subject’s head and located the C3 electrode position of the international 10–20 EEG system. The technician placed one sponge electrode over C3 and the other over the contralateral supraorbital area (which in most subjects is over the rostral right superior medial and frontal gyri) with both electrodes fixed in place with a taut fitted rubber strap. In anodal M1-tDCS, the anode was the C3 electrode, and current flowed from the contralateral supraorbital electrode to the C3 electrode. This procedure has been reported to increase motor-evoked potentials (MEPs) elicited with TMS and is termed excitatory tDCS (Nitsche and Paulus, 2000). In the cathodal arrangement, the cathode is the C3 electrode. This decreases MEPs and is termed inhibitory tDCS. During sham tDCS, we used the anodal arrangement, but ramped the current over 30 s and then ramped the current down over 30 s and repeated this current ramp up and down at the end of 20 min (Gandiga et al., 2006).

After placing the sponge electrodes, we measured the subject’s WDTs, HPTs, MPTs, and suprathreshold mechanical pain ratings. We then applied the C-HP model to the subject’s right leg and obtained pain intensity ratings every minute.

The technician applied tDCS using the Soterix Medical (New York, NY, United States) 1 × 1 tDCS platform with a current intensity of 1 mA for 20 min with two 5 cm by 7 cm sponge electrodes (current density: 0.0286 mA per cm^2^). To avoid eliciting phosphenes, we ramped the current up over 30 s at the beginning and down over 30 s at the end of the stimulation. The tDCS device was obscured from the assessor by a screen, and the cables leading to each sponge electrode were black to prevent cable color from revealing the stimulation type to the assessor. The technician started the stimulation 12 min after application of the C-HP model. We ramped the thermode temperature to the individualized target temperature 3 min after tDCS began. We removed the thermode and capsaicin cream 34 min after application. Immediately after the cessation of tDCS and removal of the capsaicin cream subjects completed Spielberger’s State-Trait Anxiety Inventory and the Situational Catastrophizing Questionnaire (Spielberger, 1983; Edwards et al., 2006). At the end of the session, we assessed side effects from the stimulation with a standardized questionnaire (Fregni et al., 2008). The questionnaire assessed the presence and severity of acute mood change, headache, neck pain, scalp pain, scalp burns, skin redness, sleepiness, tingling, or trouble concentrating. We also had the subjects complete the Short-Form of the McGill Pain Questionnaire version 2 in regard to the C-HP model they just experienced (Dworkin et al., 2009). We assessed the area of secondary hyperalgesia, suprathreshold mechanical pain ratings and pain intensity in the absence of stimulation at 15, 25, 45, and 65 min after removal of capsaicin cream. (Experimental overview: Figure 2B).

### MRI Experiment Subject Characteristics

All subjects who completed the first four sessions were offered continuing participation in the fMRI portion of the study. Of the 24 available, 16 right-handed subjects, aged 21–36, (7 females) participated in the fMRI study. The unblinded study assistant assigned each subject to receive a predetermined counter-balanced order of tDCS stimulation. One male subject completed the first MRI session and discontinued due to time commitments. After unblinding the data, we found this subject experienced the sham session. His data were included in the hyperalgesia model (see section “Group Level Analysis”).

We ensured continuing eligibility with a random urine drug screen as before. One male subject was re-enrolled after producing negative urine drug screens during MRI sessions.

We included subjects in the fMRI portion of the study, if they developed secondary mechanical hyperalgesia.

### fMRI Sessions

We assessed subjects’ continuing safety during the MRI sessions with safety questionnaires for the tDCS and the fMRI. During each fMRI session, the research technician placed the tDCS sponge electrodes as previously described. In order to visualize the placement of the sponge electrode, we placed a vitamin E capsule in the center of the electrode (Kozel et al., 2000). We then measured the Euclidean distance between the electrode and the hand knob of the motor cortex in real space.

After placing the subject into the MRI, we took a localizer scan and measured WDTs. For MRI we used a 3-T Tim Trio scanner (Siemens Medical Solutions, Malvern, PA, United States) using a 12-channel head coil with parallel imaging capability. While performing the structural MRI, we measured HPTs. The structural MRI was an MPRAGE protocol with 2.91 ms TE, 2,300 ms TR, 900 ms TI, flip angle of 9°, 176 slices, sagittal slice thickness 1.0 mm and 1.0 × 1.0-mm in-plane resolution over a 25.6-cm field of view.

Subjects experienced an event-related fMRI with a series of 27 painful mechanical stimuli consisting of 9 applications each of three forces (128, 256, and 512 mN) in a fixed order. This order of stimuli was counterbalanced within subject with an inter-stimulus interval of 13–17 s. Probe sequences for control and sensitized states were different. For functional imaging we used a gradient echo single-shot echo-planar-imaging sequence with 30 ms TE, 90° flip angle and 2,500 ms TR providing 162 T2^∗^-weighted volumes in 44 interleaved, 3 mm slices (no gap) with an in-plane resolution of 3.0 mm × 3.0 mm. The scan lasted 6 min 45 s. Subjects rated pain intensity in response to 3 applications each of the 128, 256, and 512 mN probes immediately after the fMRI scan while the subject remained in the scanner. Subjects were presented with a horizontal version of the 0–100 NRS (Greenspan et al., 2003).

We applied the C-HP model to the subject’s right leg and allowed it to incubate for 12 min. The subject was removed from the MRI scanner and remained supine on the scanner bed while the coil was unlocked, but not unplugged. We instructed the subject to remain as still as possible. An unblinded technician applied the tDCS device for anodal, cathodal or sham stimulation. The tDCS device was obscured from the assessor and cables leading to both sponge electrodes were black. The technician started the stimulation as soon as the scanner room was opened after informing the assessor where to place the electrode leads. The tDCS started 12–14 min after capsaicin application and 3 min before the temperature increased to the target.

After the 20-min stimulation, the assessor removed the electrode leads and placed the subject back into the MRI scanner while the C-HP model remained in place. Subsequent to an additional scan, and after the subject provided pain ratings during exposure to the C-HP model, the assessor entered the scanner room and removed the C-HP model. This was followed by a repeat of the weighted probe fMRI scan. We probed the area of hyperalgesic skin 1 cm outside the capsaicin exposure site. Aside from probe order, the post-capsaicin probe scan was identical to the pre-capsaicin scan. After the scan subjects again rated their pain intensity in response to the three forces of probes. After the scanning session the subject filled out the tDCS side effects questionnaire. (Experimental overview: Figure 2C).

### MRI Data Analysis

All preprocessing of the event-related fMRI scans used the afni_proc.py python script interface for Analysis for Functional NeuroImaging (AFNI). The first three volumes were automatically removed at acquisition to allow for signal equilibration. We used 3dToutcount to determine volumes where more than 10% of the timepoints in a TR were outliers^1^ Each TR was slice-time corrected and aligned to the top slice. Each functional time series was detrended and spikes quashed with 3dDespike. Before aligning the anatomical scan to the functional scan, the skull was removed using 3dSkullStrip. We subsequently used 3dAllineate via the align_epi_anat.py script to align the anatomy to the third functional volume. After this alignment, the anatomical volume was warped to Talairach atlas space and normalized to the ICBM452 brain using @auto_tlrc. We performed motion correction across the functional time series by aligning the functional volumes to the third volume using 3dVolreg. After registration, 3dAllineate applied the 12-parameter affine warping matrix determined during alignment of the anatomical to Talairach space to the registered functional volumes.

We created individualized functional masks from the registered normalized images. Estimated individual maps of white matter (WM), cerebral spinal fluid (CSF) and gray matter (GM) were created for each subject using 3dSeg. We applied spatial blurring using the iterative program 3dblurtoFWHM with a 6 mm FWHM filter within an analysis mask that excluded CSF and cortical WM. This level of spatial smoothing was consistent with the group average estimate of the smoothness of the noise in the functional time courses (actual estimates: *x* = 5.77 mm, *y* = 5.75 mm, *z* = 5.78 mm). We then scaled all voxel time courses to a mean signal intensity of 100. For application of the subject-level event-related model we included as regressors of no interest censoring of outlier volumes and volumes with motion exceeding 1 mm, signal derived from the eroded WM and CSF mask, demeaned motion parameters (motion in *x*-, *y*-, and *z*-planes and rotations about the *x*-, *y*-, and *z*-axes), and their first order derivatives. The model of interest was the event timing of the 128, 256, and 512 mN probes applied separately using a simple gamma model with a hemodynamic response function with a peak delay of 4 s using AFNI’s 3dREMLfit (Cauda et al., 2014). 3dREMLfit implements an autoregressive moving average model [ARMA(1,1)] in a restricted maximum-likelihood framework.

### Group Level Analysis

For group level analysis, we used AFNI’s linear mixed-effects modeling program 3dLME (Chen et al., 2013). We designed two separate linear mixed models (LMM): one of sensitization and one of intervention. The factors in the sensitization model were probe force (levels: 128, 256, and 512 mN) and state (levels: control and hyperalgesia) with data from sham sessions of all 16 subjects. The model of intervention effect included probe force (levels: 128, 256, and 512 mN) and stimulation (levels: anodal, cathodal, and sham) with data from scans taken after the C-HP model. For pain intensity covariation and mean BOLD response to mechanical pain, we used AFNI’s 3dttest++. We implemented a minimal voxel-wise *p*-value threshold of 0.005 for covariate and contrast analyses and 0.001 for mean BOLD response during control and hyperalgesia states. We implemented a voxel-wise threshold of 0.0001 for mean BOLD response collapsed across probe force levels for control and hyperalgesia scans. For voxel tables detailing contrasts and covariate analysis results, we implemented an initial voxel-wise threshold of 0.005, while we thresholded all other voxel tables at an initial voxel-wise threshold of 0.001. To elucidate coordinates of local maxima within large clusters of the thresholded maps, we reevaluated statistical maps after reducing the *p*-value threshold by a factor of 10 (e.g., 0.001–0.0001). We chose a minimum *p*-value threshold of 0.005 since this corresponds to a minimum false discovery proportion of 0.067 (Colquhoun, 2014).

To determine areas of significant conjoint activation across disparate spatial parametric map tests we used logical conjunction analysis (Nichols et al., 2005).

To correct for multiple comparisons, we estimated the spatial autocorrelation function of the residual noise within our analysis mask using 3dFWHMx and used the resulting function parameters and 3dClustSim to calculate cluster extent criteria (CEC) for both the 3dLME and 3dttest++ statistical maps. We restricted analysis to four anatomically based masks encompassing cortical gray matter, cerebellum, subcortical gray matter, and brainstem. The CEC for the cortical GM mask for a voxel-wise *p*-value of 0.005 was 546 mm^3^, 0.001 was 249 mm^3^, and 0.0001 was 103 mm^3^. For the cerebellum mask the CEC for a voxel-wise *p*-value of 0.005 was 327 mm^3^, 0.001 was 149 mm^3^, and 0.0001 was 57 mm^3^. For the brainstem and subcortical gray matter masks, we imposed a minimum CEC of 2 voxels in real space (54 mm^3^) for *p*-values of 0.005–0.0001.

### Region of Interest Analysis

To analyze the effect of the C-HP model and intervention type on the BOLD signal response to painful mechanical stimuli, we extracted beta estimates from subject-level analyses from five regions of interest anatomically supported by prior studies including a neuroimaging meta-analysis of probing of skin in areas of secondary mechanical hyperalgesia (Lee et al., 2008; Seifert et al., 2009; Lanz et al., 2011). We localized ROIs in the pregenual ACC [pACC: (2, 40, 17)], anterior MCC [aMCC: (-2, 32, 27)], left somatosensory cortex [L-S1: (-56, -23, 39)], medial prefrontal cortex [MPFC: (2, 59, 9)], and anatomically drawn periaqueductal gray [PAG (center of mass): (0, 29, -7)]. The ROIs for unilateral L-S1, bilateral pACC, bilateral MPFC, and bilateral aMCC were drawn as spheres of 5 mm radii. In the case of bilateral seeds, there was one 5 mm radius seed in each hemisphere. Since the evaluation of post-treatment effects in RCTs remains somewhat controversial in the clinical trial literature, we analyzed the extracted beta estimates using three different structures of linear mixed effect models (Winkens et al., 2007; Egbewale et al., 2014; Twisk et al., 2018). Following the recommendations of Egbewale et al. (2014) and Twisk et al. (2018), we modeled the post-treatment BOLD response to pinprick in the hyperalgesic area with fixed effect factors of intervention (levels: anodal, cathodal, and sham), and probe force (levels: 128, 256, and 512 mN), without a fixed effect interaction; and random effect factors of session order and probe force (and their interaction) nested within each subject while controlling the modeling of the fixed effect factors with pre-treatment BOLD responses. This model is similar to an ANCOVA but has the additional effect of controlling for individuality of session order and probe force on BOLD responses. This method of modeling the response data balances statistical bias, precision and power minimizing both types I and II error (Egbewale et al., 2014). We present these results in the main text. It is important to differentiate this analysis from the analysis conducted on post-treatment delta scores for pain intensity and area of pin prick hyperalgesia. All of those data were collected after both the capsaicin model and tDCS intervention, and therefore, within subject comparisons of anode to sham and cathode tDCS effects are not confounded by baseline pain sensitivity. To further validate this approach, we present the results of a control LMM evaluating the fixed effect factors of intervention and probe force on pre-treatment, pre-sensitization BOLD responses, with random effect factors of session order and probe force nested within subject. We additionally present the results of a full intervention by state LMM and a change score analysis (CSA) LMM on the change in BOLD response between the pre-capsaicin, pre-intervention state and the post-capsaicin, post-intervention state. All models had the same random effects factor structure, with session order, probe force and their interaction nested within subject. This replicates a repeated-measures ANOVA, which enables statistical control for any confound of session order. The CSA LMM and intervention by state LMM results are presented in Supplementary Material. All figures generated from this analysis present estimated marginal means from the associated LMMs, which were derived using the R package lsmeans (least square means), while t-stats were generated using the glht (generalized linear hypothesis test) function and ‘marginal’ *F*-stats, also known as type 3 *F*-stats, were derived using the anova function in the R Base package from each individual LMM model object. For significant effects in each model, we conducted uncorrected *post hoc* paired *t*-tests generate by the R function glht focusing on hypothesized contrasts including anodal ≠ sham, cathodal ≠ sham, anodal ≠ cathodal, and BOLD response to probing in baseline state ≠ that in sensitized state.

### Experimental Design and Statistical Analysis

We analyzed data using IBM SPSS Statistics for Windows 21.0 and R 3.2.5. We assessed normality for all data sets using the Shapiro–Wilk test. To allow parametric analyses, we found a square-root transformation [transformed data = √(original data+1)] provided the best transformation for pain ratings in response to mechanical probes. Ratings of on-going pain during the C-HP model were normally distributed. We analyzed pain intensity rating data using linear mixed models (LMMs). We derived parameter estimates using restricted maximum likelihood. The fixed effects portion of the LMMs included intervention, time and force as factors, where appropriate. Where time was a factor, the autoregressive function was modeled as a variance power function with an empirically derived exponent (Gałecki and Burzykowski, 2013).

To investigate the effect of tDCS intervention, we conducted an LMM for pain ratings provoked by painful mechanical stimuli in the area of secondary hyperalgesia. The intervention factor had 3 levels: anodal, cathodal and sham. For models evaluating pain intensity ratings, we included force as a factor with three levels 128, 256, and 512 mN.

The random effects portion of the LMMs included session order and time nested within subject. Session order with levels first, second, and third, was included to control for any order effects not controlled by randomization. Model selection was guided by Akaike’s Information Criteria (AIC) and log-likelihood comparison where factors were added progressively until model fit no longer improved. Since we hypothesized anodal tDCS would lead to a greater reduction in secondary hyperalgesia compared to cathodal or sham tDCS, we evaluated significance on contrasts of anodal versus sham and anodal versus cathodal.

We evaluated pain intensity ratings to painful mechanical stimuli while in the scanner for order effects and randomization to intervention effects using LMMs and *post hoc* paired *t*-tests where appropriate. We calculated a measure, percent hyperalgesia, which reflects the session specific effect of the C-HP model on pain ratings. *Percent Hyperalgesia* = 100 × $\frac{PI_{\mathrm{PostCHP}}-PI_{\mathrm{Pre}\mathrm{CHP}}}{100-PI_{\mathrm{Pre}\mathrm{CHP}}}$. Where *PI*_PostCHP_ is the pain intensity reported in response to painful mechanical stimuli in the sensitized area after exposure to the C-HP model and *PI*_PreCHP_ is the pain intensity reported in response to painful mechanical stimuli on the leg before exposure to the C-HP model. Additionally, we analyzed the change in area of hyperalgesia for subjects who completed the neuroimaging study (*n* = 15).

## Results

### RCT Sessions

#### Measures of Secondary Hyperalgesia: Pain Intensity

The initial LMM on pain intensity ratings to mechanical probes (at 128, 256, and 512 mN), at 15, 25, 45, and 65 min after capsaicin-heat pain model, revealed a significant intervention effect (*F* = 3.6; *p* = 0.028), and a significant intervention by time interaction (*F* = 4.22; *p* = 0.015). Specifically, there was a significant anode versus sham intervention effect (*t*-stat = -2.61; *p* = 0.009) and a trend of an anode versus cathode effect (*t*-stat = -1.86; *p* = 0.063). Additionally, there was a significant anode versus sham by time interaction (*t*-stat = 2.9; *p* = 0.004), but no anode versus cathode by time interaction (*t*-stat = 1.2; *p* = 0.23).

To establish that pain intensity ratings taken at 15 min after removal of the C-HP did not differ among intervention types, we used an LMM including intervention type (anodal, cathodal, or sham tDCS) and probe force (128, 256, and 512 mN) and found no significant intervention main effect (*F*-stat = 2.08; *p* = 0.13). There was a significant main effect of probe force (*F*-stat = 52.1; *p* < 0.0001). This analysis at the first timepoint allows us to consider this timepoint a baseline and to analyze the change over time for each intervention separately to determine if there is a more rapid resolution of hyperalgesia pain intensity with one treatment compared to the others.

**FIGURE 3:**
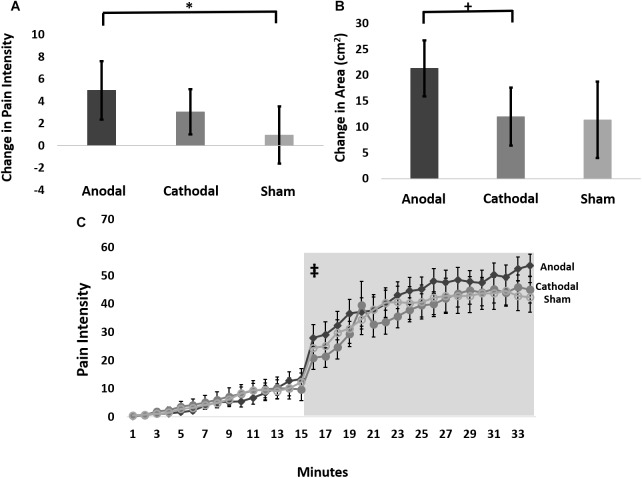
**(A)** Average change in pain intensity (delta scores) from 15 to 65 min grouped by intervention type and collapsed across probe force (128, 256, and 512 mN) (^∗^ anodal > sham *p* = 0.02). **(B)** The change in area of pinprick hyperalgesia (delta score) from 15 to 65 min grouped by intervention type (+ anodal > cathodal *p* = 0.075). **(C)** Time course of pain ratings during capsaicin mediated heat allodynia. Anodal (dark gray), cathodal (medium gray), or sham tDCS (light gray) began 12 min after capsaicin application, and the thermode temperature ramped up to the individualized target temperature at 15 min after capsaicin application. Light gray box demarcates post-treatment time course where pain intensity ratings were significantly higher during anodal compared to sham stimulation. ‡ = anodal > sham stimulation *p* = 0.0009. Error bars are SEM.

Therefore, we further analyzed these data using an LMM for the change in pain ratings between the 15-min timepoint and the 65-min timepoint post-capsaicin exposure, which we term delta scores. This analysis revealed a trend intervention effect (*F*-stat = 2.8; *p* = 0.06), but no effect of probe force (*F*-stat = 0.16; *p* = 0.69). Pairwise comparisons of delta scores (collapsed across probe weight) revealed that anodal tDCS reduced the pain intensity to probing in the hyperalgesic zone to a significantly greater extent than sham (*t*-stat = -2.4, *p* = 0.02), but not cathodal tDCS (*t*-stat = -1.4, *p* = 0.15) (Figure 3A). The effect size for the intervention effect comparing anodal to sham tDCS was 0.3, putting this effect into the small to medium range (Fritz et al., 2012).

#### Measures of Secondary Hyperalgesia: Area of Secondary Hyperalgesia

The overall LMM for area of secondary hyperalgesia showed a significant anode versus cathode by time interaction (*t*-stat = 2.1; *p* = 0.04) and a trend for the anode versus cathode contrast (*t*-stat = -1.8; *p* = 0.076). However, the anode versus sham contrast (*t*-stat = -0.11; *p* = 0.91) and anode versus sham contrast by time interaction (*t*-stat = 0.19; *p* = 0.85) were not significant. The LMM revealed a significant effect of time (*t*-stat = -4.95; *p* < 0.0001). Evaluating the factors separately within an ANOVA framework, we derived *F*-stats from the LMM for intervention (*F*-stat = 2.05; *p* = 0.13), time (*F*-stat = 24.5; *p* < 0.0001) and intervention by time interaction (*F*-stat = 2.6; *p* = 0.075).

To establish that the area of secondary hyperalgesia measured 15 min after removal of the C-HP did not differ among intervention types, we used an LMM for intervention type (anodal, cathodal, or sham tDCS) found no significant main effect of intervention (*F*-stat = 0.78; *p* = 0.46). This analysis at the first timepoint allows us to consider this timepoint a baseline and to analyze the change over time for each intervention separately to determine if there is a more rapid reduction of secondary hyperalgesia area with one treatment compared to the others.

Therefore, we performed an LMM analysis on delta scores of areas of secondary hyperalgesia contrasts of which revealed that anodal tDCS produced a trend for greater reduction in the area of secondary hyperalgesia during the post-stimulation period compared to cathodal stimulation (*t*-stat = -1.82; *p* = 0.075), and a weaker, though qualitatively similar effect compared to sham stimulation (*t*-stat = -1.57; *p* = 0.12) (Figure 3B). The effect size for the intervention effect comparing anodal tDCS to cathodal tDCS was 0.35 and the effect size for the comparison of anodal tDCS to sham tDCS was 0.33, placing the magnitude of both of these effects in the small to medium range.

#### Intervention Effect on Thermal Allodynia

The LMM revealed a significant tDCS intervention effect on pain intensity ratings when contrasting anodal versus sham intervention collected during simultaneous exposure to the C-HP model especially after the initiation of stimulation. When evaluating the entire time course, anode versus sham (*t*-stat = 3.7; *p* = 0.0002) and anode versus cathode (*t*-stat = 2.28; *p* = 0.023) contrasts were significant (Figure 3C). Additionally, the anode versus sham contrast by time interaction was significant (*t*-stat = -5.5; *p* < 0.0001) and the anode versus cathode contrast by time interaction was significant (*t*-stat = -4.6; *p* < 0.0001). Due to the increase in pain intensity ratings during exposure to the C-HP model the time factor was highly significant (*t*-stat = 9.5; *p* < 0.0001). This led us to evaluate the pre- and post-intervention pain intensity rating time courses separately. During the pre-intervention time period neither the anode versus sham (*t*-stat = 0.74; *p* = 0.46) nor anode versus cathode (*t*-stat = 0.14; *p* = 0.89) contrasts were significant, and neither intervention by time interaction was significant (*t*-stat < -0.70; *p* > 0.48). In contrast, after initiation of stimulation both the anode versus sham contrast (*t*-stat = 3.3; *p* = 0.0009) and the anode versus sham by time interaction (*t*-stat = -3.0; *p* = 0.003) were highly significant. This resulted from an increase in pain ratings in response to heat allodynia induced by the C-HP model during anodal compared to sham tDCS. In contrast, neither the anode versus cathode contrast (*t*-stat = 0.17; *p* = 0.86) nor the anode versus cathode by time interaction (*t*-stat = -0.02; *p* = 0.98) were significant. The time factor was significant during both pre-intervention (*t*-stat = 5.20; *p* < 0.0001) and post-intervention (*t*-stat = -5.1; *p* < 0.0001) time periods.

The LMM evaluating ongoing pain intensity ratings after C-HP model removal revealed no significant anode versus sham (*t*-stat = 1.15; *p* = 0.25) or anode versus cathode (*t*-stat = -0.15; *p* = 0.88) contrast or intervention contrast by time interaction effects (*t*-stat < 0.50; *p* > 0.62). Nonetheless, pain intensity ratings after removal of the C-HP model decreased significantly over time (*t*-stat = -5.54; *p* < 0.0001).

#### Side Effect Profile

The LMM analysis found no significant differences among anodal, cathodal or sham stimulations in reported side effects.

### fMRI Sessions

#### Psychophysics and Questionnaires

The subset of subjects participating in the MRI portion of the study did not significantly differ in age, trait anxiety, warmth, heat pain or mechanical pain sensitivity, sex ratio, or any measure of mechanical hyperalgesia in response to the C-HP model (Supplementary Table S2). Furthermore, these subjects experienced a greater reduction in mean area of secondary hyperalgesia after anodal tDCS (19 cm^2^) compared to cathodal (9 cm^2^) or sham (4 cm^2^) stimulation, although this difference did not reach statistical significance (anodal versus sham *t*-stat = -1.8, *p* = 0.08; anodal versus cathodal *t*-stat = -1.5, *p* = 0.15). The effect size for these intervention effects were 0.5 for anode versus sham and anode versus cathode contrasts. The mean increase in mechanical pain after hyperalgesia developed was less with anodal (9% increase) or cathodal tDCS (9%) compared to sham stimulation (14%), although this difference did not reach statistical significance (anodal versus sham *t*-stat = 1.25, *p* = 0.23; cathodal versus sham *t*-stat = 1.79, *p* = 0.096; anodal versus cathodal *t*-stat = 0.033, *p* = 0.97). The effect size for the anodal versus cathodal intervention effect was 0.01, the anodal versus sham invention effect was 0.3, and the cathodal versus sham intervention effect was 0.4. The LMM evaluating the intervention effect on heat allodynia pain intensity ratings in the MRI environment revealed no significant effect in the anode versus cathode (*t*-stat = -0.43, *p* = 0.67) or the anode versus sham (*t*-stat = -1.28, *p* = 0.20) contrast or either interaction with time (*t*-stat < 0.18, *p* > 0.85).

RM-ANOVA analyses evaluated the intervention effect on STAI-S, SCQ, SFMPQ2 sum and subscale scores revealed no significant effect (all *p*-values > 0.16).

#### Accuracy of Position of C3 Electrode Targeting M1

The average skull-to-cortex distance in the 16 subjects in the area of the motor cortex was 16.3 mm (range: 12–22 mm). The average distance from the center of the fiducial to the cortical surface of the motor cortex was 33.7 mm (range: 26.2–47.4 mm), giving the average distance from the center of the fiducial to the scalp surface 17.6 mm (range: 9.2–31.4 mm). This average displacement is within the distance of the size of the electrode (50 by 70 mm). There was no significant effect of intervention on electrode distance from the target (*F* = 1.1; *p* = 0.35).

#### Central Sensitization Modulates BOLD Response to Painful Mechanical Stimuli

**FIGURE 4:**
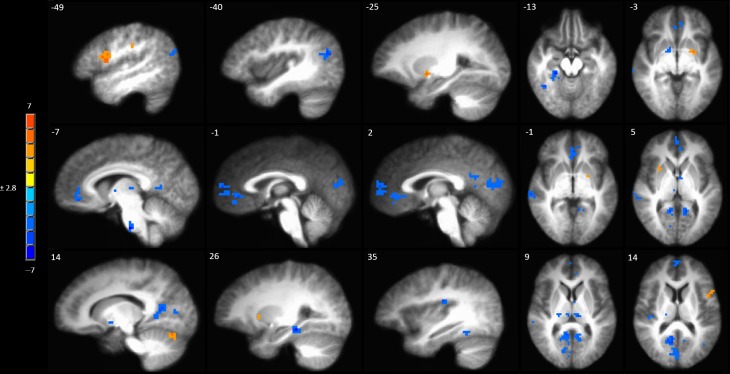
Contrast map of mechanical pain-related activation during the sham tDCS session where post-C-HP > pre-C-HP. Maps are voxel-wise cluster extent corrected threshold of *p* ≤ 0.005. Coordinates are according to the Talairach atlas.

A full brain voxel-wise linear mixed model contrast comparing the baseline BOLD response to mechanical pain to the BOLD response to sensitized mechanical pain after sham tDCS revealed multiple significant clusters. Areas of significantly lower BOLD response evoked by mechanical pain in the hyperalgesic zone included the bilateral middle temporal gyrus, posterior cingulate cortex (PCC) bilaterally, left pACC and sACC, right parahippocampal gyrus, fusiform gyrus, posterior insula, medial prefrontal cortex (MPFC) (Figure 4 and Supplementary Table S3). Subcortically significant reductions in BOLD response to painful mechanical stimuli included left caudate, right medial globus pallidus, left medial dorsal thalamus and an area in the right thalamus (Supplementary Table S3). The model also revealed cortical areas where painful mechanical stimuli after induction of sensitization evoked greater BOLD responses including the left inferior frontal gyrus, pre- and postcentral gyrus. Subcortical regions of increased BOLD response after sensitization included the right cerebellum, left putamen, and ventral pons.

#### Anodal Motor Cortex tDCS Opposed the Neurophysiological Effects of Sensitization With in the DPM Network

**FIGURE 5:**
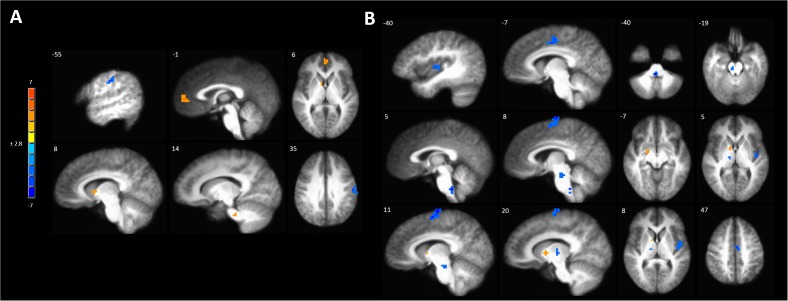
Activations to painful mechanical stimuli in the hyperalgesic zone were modulated by tDCS. **(A)** Anodal compared to sham tDCS (red, anodal > sham, blue sham > anodal); **(B)** Cathodal compared to sham tDCS (red, cathodal > sham, blue sham > cathodal). Maps are voxel-wise threshold of *p* ≤ 0.005, cluster extent corrected. Coordinates are according to the Talairach atlas.

Anodal tDCS targeted to the left M1 significantly increased BOLD responses in the left MPFC, right caudate and pontine nuclei and reduced BOLD response in the left precentral gyrus evoked by painful mechanical stimuli in hyperalgesic skin compared to sham stimulation (Figure 5A and Supplementary Table S4A).

Cathodal M1 tDCS significantly reduced BOLD responses in the right dorsomedial frontal cortex in the supplementary motor area (SMA) and the left MCC and posterior insula to painful mechanical stimuli after sensitization compared to sham stimulation (Figure 5B and Supplementary Table S4B). Within the brainstem significantly reduced BOLD responses were found in the rostral medulla and rostral pons. Increased BOLD responses after cathodal tDCS compared to sham stimulation were found in the right medial globus pallidus and caudate nucleus.

**FIGURE 6:**
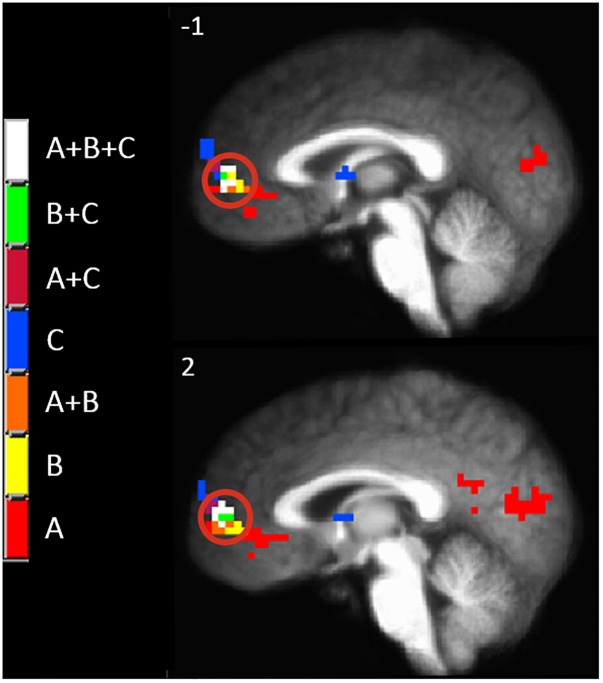
The MPFC, indicated by the red circle, was the only region specifically modulated by anodal tDCS and the C-HP model. The functional anatomical overlap of (A) negative activation to painful mechanical stimuli in the hyperalgesic zone after sham tDCS (B) activation to painful mechanical stimuli in hyperalgesic zone where anodal > sham tDCS, and (C) where activation to painful mechanical stimuli before capsaicin exposure is significantly greater than after exposure [cluster-extent corrected voxel-wise threshold *p* ≤ 0.005 (B and C) or *p* < 0.001 (A)].

Since there appeared to be a functional anatomical convergence between three effects of interest in the MPFC, we created an overlap map including: the negative part of the BOLD response to painful mechanical stimuli (CEC at *p* < 0.001), areas of evoked BOLD response to mechanical pain after the C-HP model and anodal stimulation greater than that after C-HP and sham stimulation (CEC at *p* < 0.005), and areas of evoked BOLD response to mechanical pain which are greater during pre-C-HP than after C-HP model (CEC at *p* < 0.005) (Figure 6). This revealed an area of overlap in the MPFC anterior to the pACC in Brodmann area 10.

**FIGURE 7:**
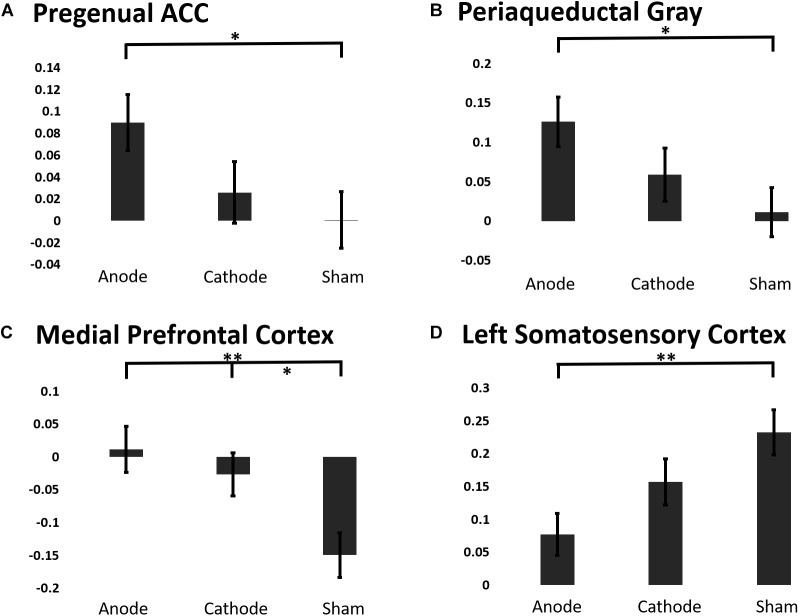
The estimated marginal means of a region of interest analysis of post-treatment BOLD response linear mixed effects model controlling for baseline BOLD response to painful mechanical stimuli from ROIs in the **(A)** pACC, **(B)** PAG, **(C)** MPFC, **(D)** Left S1 (^∗∗∗^*p* ≤ 0.001, ^∗∗^*p* ≤ 0.01, ^∗^*p* ≤ 0.05).

To elucidate the effects of anodal or cathodal tDCS on BOLD responses in the descending pain modulatory network, we extracted the beta coefficients from each intervention session before and after the C-HP model (Figure 7). The analysis focused on the pACC, aMCC, PAG, and MPFC anterior to the pACC. Additionally, we included L-S1 adjacent to the stimulated motor cortex and contralateral to the area of sensitized skin. The LMM evaluating the fixed effect factors of treatment and force controlling for pre-treatment responses found significant effects of intervention for the extracted BOLD response in the pACC (*F*-stat = 6.73, *p* = 0.011), PAG (*F*-stat = 8.19, *p* = 0.0050), MPFC (*F*-stat = 12.47, *p* = 0.0006), and L-S1 (*F*-stat = 5.63, *p* = 0.019) (Figure 7). Furthermore, this analysis found significant effects of probe force in the PAG (*F*-stat = 8.59, *p* = 0.0041).

Contrary to our predictions evoked BOLD responses in aMCC showed no consistent effect of intervention, (post-treatment LMM: *F*-stat = 2.00, *p* = 0.16). *Post hoc* analysis showed that anodal tDCS prevented the reduction in the BOLD response in the PAG and pACC evoked by painful mechanical stimuli in the hyperalgesic zone. Finally, both anodal and cathodal tDCS blocked the negative evoked BOLD response to mechanical pain in the MPFC induced by the C-HP model (Figure 7).

The LMM analyzing any potential confound effect of baseline BOLD responses to painful mechanical stimuli in normal skin before capsaicin or tDCS exposure found no significant effect of intervention in the aMCC, pgACC, PAG, MPFC, or L-S1 (Supplementary Table S5C).

The results of both the intervention by state LMM (Supplementary Table S5A and Supplementary Figure S1) and change score analysis LMM (Supplementary Table S5B and Supplementary Figure S2) supported the results of the post-treatment LMM in the MPFC, L-S1, and aMCC, but not the pgACC and PAG. This echoes the previously reported artefactually conservative bias inherent in these models (Egbewale et al., 2014; Twisk et al., 2018).

#### Anodal tDCS Abolishes Pain Intensity Covariance With BOLD Response in Pain Responsive Areas

**FIGURE 8:**
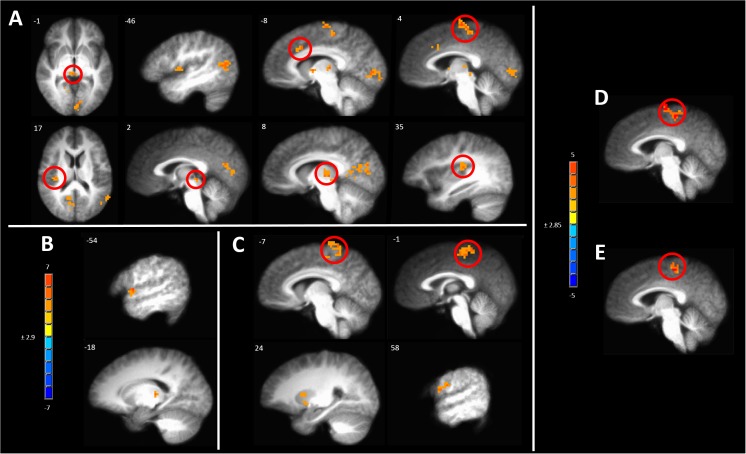
Significant correlation of pain intensity with BOLD response evoked by painful mechanical stimuli after **(A)** sham, **(B)** anodal or **(C)** cathodal tDCS. **(D)** Conjunction analysis between the covariance maps of pain intensity with BOLD response in the pre-capsaicin condition following sham stimulation condition revealed coincident correlation in the paracentral lobule. **(E)** A similar analysis between the sham and cathodal stimulation conditions revealed coincident correlation only in the paracentral lobule. The resultant conjoint map of sham and anodal stimulation conditions revealed no such overlap. Maps are voxel-wise threshold of *p* ≤0.005, cluster extent corrected. Coordinates are according to the Talairach atlas.

One way to identify regions of the brain involved in processing of subjective sensations such as pain is to correlate the BOLD response evoked by the stimulus to the magnitude of the intensity of the sensation (Coghill et al., 1999). After induction of hyperalgesia during sham treatment, mechanical stimuli led to a pattern of BOLD response that correlated with pain intensity which included several pain-responsive regions such as the posterior insula, paracentral lobule (PCL), and aMCC (Figure 8A and Supplementary Table S5A). Additional areas of positive correlation included the superior temporal gyrus in the area of the temporoparietal junction, the ipsilateral PCC, and medial dorsal thalamus (Figure 8A and Supplementary Table S5A).

In *contrast*, all areas of positive correlation of evoked BOLD response with pain intensity present after sham stimulation were abolished when hyperalgesia induction was accompanied by anodal tDCS leaving significant clusters only in the contralateral thalamus and lip of the frontal operculum (Figure 8B and Supplementary Table S5B).

When induction of hyperalgesia was accompanied by cathodal tDCS areas of positive correlation of evoked BOLD response with pain intensity remained in the bilateral putamen and the PCL and postcentral gyrus in the somatotopic leg area (Figure 8C and Supplementary Table S5C). To verify the spatial correlation and statistical strength of these changes in pain intensity-evoked BOLD response correlations, we conducted conjunction analyses between baseline and post-CHP states after sham stimulation, post-CHP state after sham stimulation and cathodal tDCS and post-CHP state after sham stimulation and anodal tDCS. When applying a conservative CEC we found significant overlap between baseline and sham conditions and between cathodal and sham condition in left PCL and medial SFG (Figure 8D,E). There were no significant clusters as a result of the conjunction analysis between sham and anodal conditions. Thus, anodal tDCS was associated with an absence of significant pain intensity covariation in brain-wide BOLD response, whereas numerous clusters of pain-intensity BOLD covariation were found during mechanical pain after sensitization after sham or cathodal stimulation.

## Discussion

We determined if a single session of anodal M1 tDCS would significantly reduce sensitization-induced hyperalgesia and allodynia; then probed the neurophysiological effects of anodal tDCS in healthy adults. We predicted psychophysical measures of central sensitization would be more reduced by anodal compared to sham or cathodal tDCS, consistent with the efficacy of M1 neuromodulation in pain syndromes driven by central sensitization (Fregni et al., 2006; Woolf, 2011). Anodal tDCS resulted in greater reduction in pain intensity from probing in the hyperalgesic zone than sham tDCS after C-HP removal. While the reduction in pain intensity from probing in the hyperalgesic zone was greater after anodal compared to cathodal stimulation, this difference did not reach statistical significance. Anodal tDCS produced a trend toward greater reduction in the area of secondary hyperalgesia compared to cathodal tDCS. For the reduction in area of secondary hyperalgesia, we found similar effect sizes comparing anodal to cathodal tDCS (Cohen’s *d* = 0.35) and to sham tDCS (Cohen’s *d* = 0.33), despite an absence of statistical significance.

In probing the neurophysiological mechanism of M1-anodal tDCS, we found sensitization blunted pain-related activity in pACC, PAG, and MPFC, areas implicated in pain modulation. Anodal tDCS normalized these BOLD responses to baseline levels. Compared to cathodal or sham, anodal tDCS suppressed BOLD activity in S1, which was enhanced by sensitization. Analysis of the covariation of pain intensity with BOLD response to mechanical stimuli revealed anodal tDCS disrupted the brain-wide correlation of subjective sensation with evoked BOLD response. Together these results support the hypothesis that, even after one session, M1-anodal tDCS modulates activity in primary nociceptive processing and inhibitory areas of the brain, consistent with pain reduction.

Numerous studies have evaluated the effect of non-invasive cortical neuromodulation on acute pain in healthy subjects (Mylius et al., 2012; Vaseghi et al., 2014). However, we know of only six studies investigating the effect of non-invasive neuromodulatory techniques on persistent or repetitive pain models in healthy subjects. Only two of these studies targeted M1 (Tamura et al., 2004; Ihle et al., 2014), while others targeted the DLPFC (Fierro et al., 2010; Taylor et al., 2013; Lin et al., 2017; Seminowicz et al., 2018).

Ihle and colleagues found no effect of anodal or cathodal M1-tDCS on pain intensity ratings in response to repeated 48°C heat stimuli, consistent with our negative result in regard to heat allodynia. Neither the present study nor that of Ihle and colleagues found a significant analgesic effect of M1 anodal tDCS on pain ratings during induction of primary hyperalgesia. In fact, our study revealed anodal tDCS increases pain ratings to evoked thermal allodynia.

The effects of M1 neuromodulation on provoked pain in healthy subjects are inconsistent (Mylius et al., 2012; Aslaksen et al., 2014; Vaseghi et al., 2014). For example, while no studies have found a significant effect of M1 targeted neuromodulation on HPTs, Aslaksen and colleagues found anodal M1 tDCS reduced suprathreshold heat pain ratings specifically to a 47°C stimulus (Bachmann et al., 2010; Borckardt et al., 2011; Jürgens et al., 2012). A lack of efficacy of M1 tDCS on heat-evoked pain is consistent with our results during heat allodynia. CNS processing of heat-evoked pain and heat allodynia, while clearly different at some level, is largely overlapping, considering primary afferent sensitization is likely responsible for capsaicin-induced heat allodynia (Baumann et al., 1991; LaMotte et al., 1991; Simone et al., 1991). What divides the mechanism of central sensitization from primary afferent sensitization in the C-HP model is enhanced responsiveness of spinal dorsal horn neurons to mechanical stimulation in the hyperalgesic zone. The present findings suggest M1 tDCS may selectively influence higher-order neuronal circuitry changed as a result of central sensitization.

### A Model of M1 Neuromodulation Involving the Descending Pain Modulatory Network

Several studies have demonstrated the involvement of the DPM network in response to epidural motor cortex stimulation (EMCS) (Peyron et al., 2007; Pagano et al., 2011, 2012). Our study is the first to extend these findings to non-invasive neuromodulation targeting M1 on processing of painful mechanical stimuli. We found excitatory anodal tDCS modulated BOLD responses to hyperalgesic painful stimuli in several brain regions including MPFC, pACC, PAG and brainstem at the level of the pontine reticular formation. Studies of EMCS in animal models of chronic pain have demonstrated increased activity relative to sham treated animals in the ACC and PAG (Kudo et al., 2014). Human neuroimaging studies have found similar results in the pACC and PAG (García-Larrea et al., 1999). Further, PET studies of EMCS in chronic pain patients found reduced binding potential of opioid ligand in response to EMCS in aMCC and PAG (Maarrawi et al., 2007). Findings in the PAG of animals have been mixed, with some studies showing an increase in activity, a decrease in activity, and one study finding either an increase or decrease in activity depending on the side relative to MCS (França et al., 2013; Dimov et al., 2016). Given the functional heterogeneity of the PAG and inability of PET or traditional fMRI studies in humans to resolve the subregions of the PAG, it is likely different subregions respond to M1 neuromodulation in a specific manner (Behbehani, 1995; Linnman et al., 2012). We found increased BOLD responses in the brainstem at the level of the pontine reticular formation to painful stimuli specifically after M1-anodal tDCS. Due to the size of brainstem nuclei, we are unable to confirm the precise anatomical location of this cluster, but studies in animals and humans have shown modulation of this region in response to EMCS.

We found reduced evoked BOLD in the paracentral lobule (PCL) corresponding to the somatotopic leg area in response to painful mechanical stimuli. Studies in animal models have demonstrated both decreased neuronal spiking and decreased BOLD response after EMCS in S1 (Chiou et al., 2012; Jiang et al., 2014). Additionally, excitatory M1 rTMS causes reorganization of somatosensory evoked potentials and reduction in the amplitude of painful laser evoked potentials (LEPs) (de Tommaso et al., 2010; Lefaucheur et al., 2010; Houze et al., 2013). Together these findings suggest modulation of S1 excitability by neuroplastic modulation mediated through corticocortical pathways. An interaction between M1 excitability and pain perception and processing is found both in chronic pain patients and tonic pain in healthy subjects (Valeriani et al., 1999; Lefaucheur et al., 2006; Antal et al., 2010; Fierro et al., 2010; Parker et al., 2016; Schabrun et al., 2016). Aberrant motor cortex excitability induced by tonic pain in healthy subjects or by chronic pain in patients is normalized by analgesic M1 neuromodulation. The effects of tDCS on motor cortex excitability are sensitive to electrode placement and montages that target S1 have little effect on motor cortex excitability or pain (Nitsche and Paulus, 2000; Vaseghi et al., 2015).

In assessing covariation of pain intensity with brain-wide BOLD response, we found several significant regions of covariation after sham tDCS and induction of central sensitization. Significant clusters of positively correlated BOLD response with pain intensity appeared in the bilateral medial thalamus, aMCC, PCL, and left temporoparietal junction. After sensitization and cathodal tDCS several areas of covariation of pain intensity with BOLD response to mechanical pain remained including left PCL, SMA, and bilateral putamen. In contrast, after sensitization and anodal tDCS few areas of BOLD response to mechanical pain covaried with pain intensity, and none survived a conjunction analysis with the sensitized mechanical pain BOLD response pain intensity covariation map (Figure 8). We interpret this finding as evidence for disruption of mechanical pain processing after M1-anodal tDCS. Interestingly, magnitude of LEPs correlates with pain intensity reported by the subjects before excitatory M1-rTMS, and M1-rTMS abolishes this relationship (Lefaucheur et al., 2010).

### Limitations

Although we found statistically significant effects of anodal tDCS compared to cathodal or sham stimulation in measures of mechanical hyperalgesia, the magnitude of differences were small. The magnitude of pain intensity reduction to probing in the sensitized zone, while statistically significant would not be considered clinically significant (e.g., 7/100 NRS point difference for the 256 mN probe). Small magnitude effects should be expected following a single tDCS session, since most therapeutic studies of M1 tDCS demonstrate the need for multiple treatment sessions for efficacy (Fregni et al., 2006; Antal et al., 2010; Luedtke et al., 2012). The strength of our design and results are that only one session was required to achieve a significant analgesic result.

Further it is important to note that blinding and similarity of sham experiences to anodal and cathodal tDCS remain problems in the conduct of tDCS studies (Gandiga et al., 2006; Brunoni et al., 2011; Russo et al., 2013). One weakness of our design is that we did not force the subjects to guess what intervention they had just received at each session and have them judge their confidence in that guess as is now common in tDCS studies (Russo et al., 2013). However, we note that we captured Likert scale ratings of various sensation typically associated with active tDCS (e.g., itching, burning) as well as perceptual stimuli that may have unblinded the sensory assessments (e.g., skin redness) and found that none of these sensations or perceptions were associated with verum compared to sham stimulation.

Since this was a within-subjects design, it may be argued that there could be carryover effects of the tDCS from one session to another session. We mitigated the potential interference of carryover effects by both imposing a session-to-session interval of 2 weeks and by adding the order the sessions occurred to the factor structure of the LMMs. Therefore, we used both experimental and statistical methods to mitigate the effect of any carryover effects on the results.

An additional limitation of using the C-HP model is that it is likely the motor cortex stimulation in chronic pain patients has the potential to target thalamic and cortical neuroplasticity resulting from chronic pain processes, such as those found in chronic pain subsequent to spinal cord injury and trigeminal neuralgia (Gustin et al., 2011, 2012; Henderson et al., 2011). Interestingly, recent results from our lab demonstrate that the C-HP model may, after persistent pain durations of 20 min, initiate neuroplastic processes previously uniquely associated with neuropathic pain [e.g., shifts to lower peak alpha in the EEG spectrum (Sarnthein, 2005; Furman et al., 2018)].

## Conclusion

Our results demonstrate a moderate effect of a single session of anodal M1 tDCS on reducing secondary hyperalgesia compared to cathodal or sham tDCS. In contrast, anodal, cathodal and sham tDCS had similar effects on measures related to primary hyperalgesia. These results suggest that if anodal tDCS is effective in ameliorating chronic pain, it should be most efficacious in disorders involving central sensitization. Using an assessor-blind randomized controlled study to investigate the neurophysiological correlates of anodal M1 tDCS in a pain model in healthy humans, we show enhanced activation of opioid-releasing brain areas such as the MPFC, pACC, and PAG, providing evidence of M1 tDCS engaging this well-established antinociceptive system.

## Ethics Statement

All subjects gave written informed consent in accordance with the Declaration of Helsinki. The protocol was approved by the Institutional Review Board, University of Maryland, Baltimore.

## Author Contributions

TM and MK designed, collected, analyzed, and interpreted the data, prepared the figures, and revised and approved the final version of the manuscript. SK collected and analyzed the data and approved the final version of the manuscript. RG designed, revised, and approved the final version of the manuscript. DS and JG designed and interpreted the data, and revised and approved the final version of the manuscript.

## Conflict of Interest Statement

The authors declare that the research was conducted in the absence of any commercial or financial relationships that could be construed as a potential conflict of interest.

## Footnotes

1 afni.nimh.nih.gov/pub/dist/doc/program_help/3dToutcount.html
