# Supplementary material for: Non-invasive Motor Cortex Neuromodulation Reduces Secondary Hyperalgesia and Enhances Activation of the Descending Pain Modulatory Network
Source: Front Neurosci. 2019 May 8;13:467. doi: 10.3389/fnins.2019.00467 (PMC6519323; doi:10.3389/fnins.2019.00467)
Supplement: Supplementary file 1 [file Data_Sheet_1.PDF]

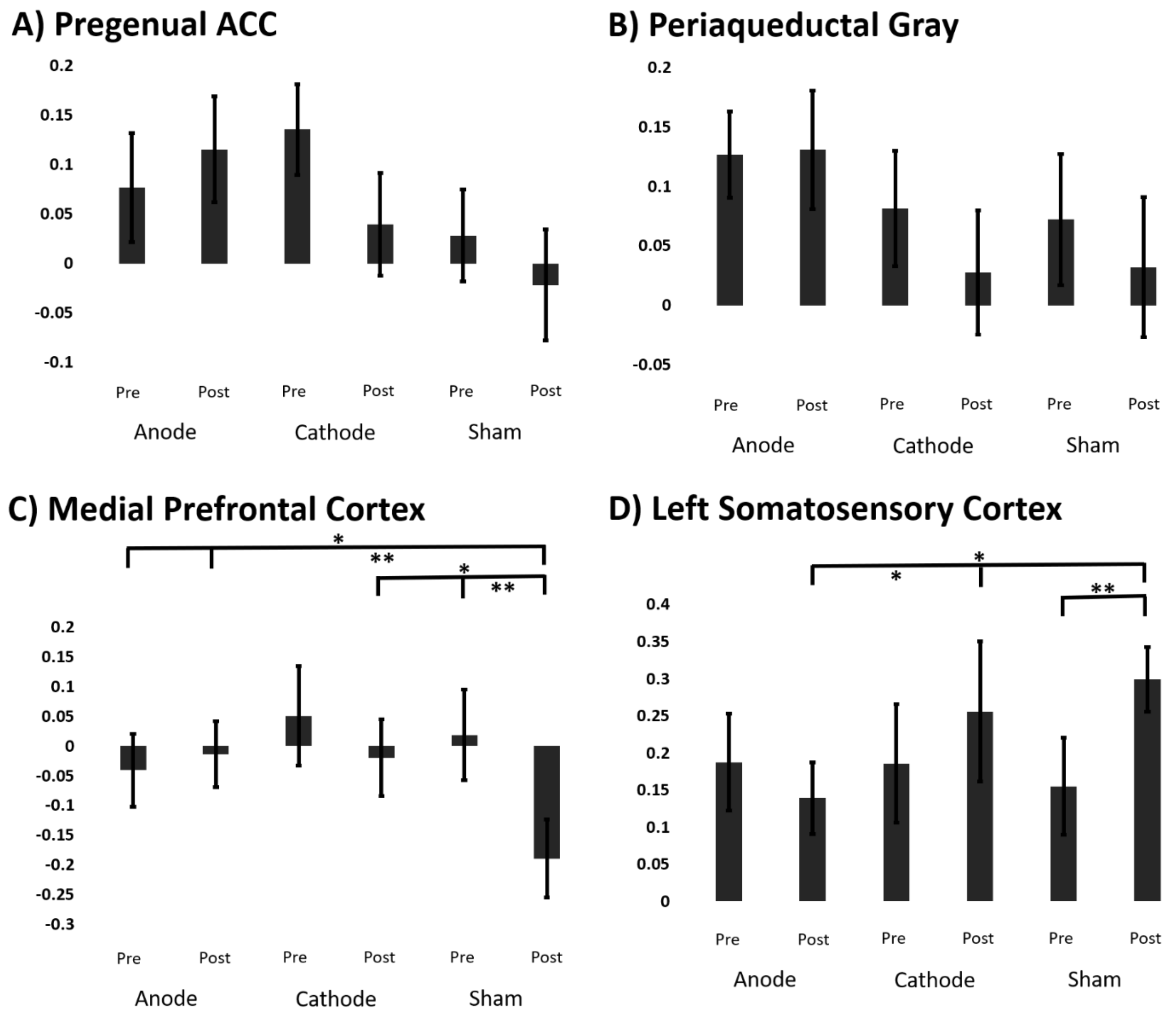

Supplemental Figure 1. *The estimated marginal means of a region of interest analysis of pre-treatment and post-treatment linear mixed effects model of BOLD response from ROIs in the (A) pACC (B) PAG (C) MPFC (D) Left S1 (** $p \leq 0.01$; * $p \leq 0.05$).*

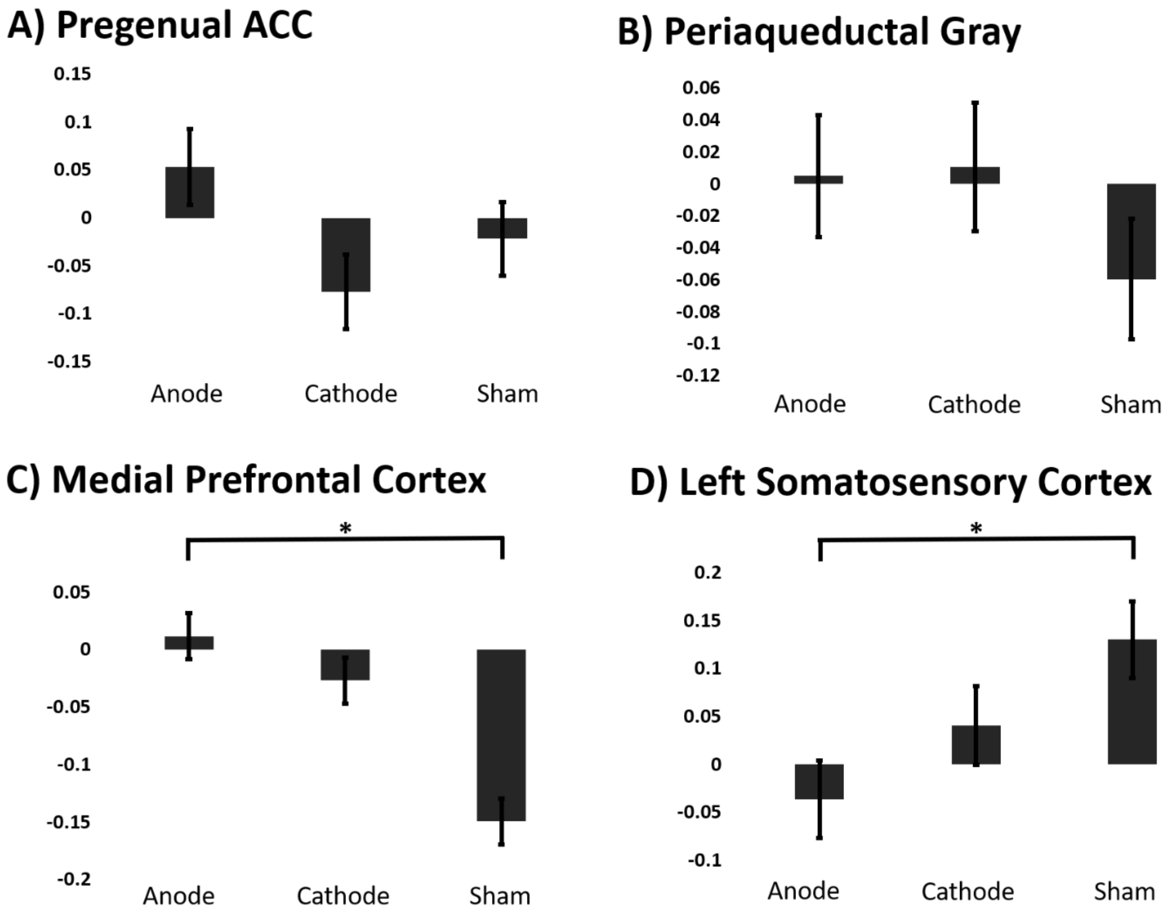

Supplemental Figure 2. *The estimated marginal means of a region of interest analysis of change score analysis of BOLD response linear mixed effects model from ROIs in the (A) pACC (B) PAG (C) MPFC (D) Left S1 (* $p \leq 0.05$*).*

Supplemental Table 1. Subject eligibility criteria.

Inclusion Criteria	Exclusion Criteria
18 to 44 years of age	Personal or family history of epilepsy
Right-handed	History of head injury in the past 12 months with a period of unconsciousness
Fluent in written and spoken English	Skin disease under the electrode site
Developed thermal allodynia (NRS rating >30 of 100) on exposure to capsaicin	Chronic pain conditions
Developed static mechanical hyperalgesia after exposure to capsaicin	Psychiatric illness requiring hospitalization in the last 12 months
Opiate-free	Methemoglobinemia
Barbiturate-free	Uncontrolled or unstable hypertension
Free of illicit drugs	Clinically significant cardiovascular disease
	Cardiac, renal, hepatic or pulmonary dysfunction
	Current or history of active malignant cancer in the past 5 years
	Hypersensitivity to capsaicin
	Multiple chemical hypersensitivity
	Ferrous metal implants
	Metallic skull plates or implants
	Implanted electronic devices
	Claustrophobia
	Cognitive impairment that interfered with obtaining consent
	Travel plans that would interfere with their study participation
	Taking centrally acting calcium or sodium channel blockers
	Taking dextromethorphane
	Taking class 2 dopamine antagonists
	Taking memantine
	Taking class I or III antiarrhythmic compounds

Supplemental Table 2. Comparison of characteristics of primary randomized control trial (RCT) subjects to MRI phase subjects.

Characteristic	RCT Phase (mean)	MRI Phase (mean)	Comparison Statistic	p-value
n =	27	15		
Females	11	7		
Males	16	8	X^2 = 0.14	0.71
Age	25	26	t = 0.89	0.38
Trait Anxiety	30	30	t = 0	1.00
Warmth Detection Threshold	36.9°C	36.9°C	t = 0	1.00
Heat Pain Threshold	43.3°C	43.4°C	t = 0.12	0.91
Mechanical Pain Threshold (MPT)	197 mN	214 mN	t = 0.59	0.56
Subjects with MPT>512 mN	4 of 15	8 of 27	X^2 = 0.041	0.84
Precapsaicin Suprathreshold Mechanical Pain Rating 64 mN	2	1.8	t = 0.17	0.86
Precapsaicin Suprathreshold Mechanical Pain 128 mN	5.8	6.5	t = 0.29	0.77
Precapsaicin Suprathreshold Mechanical Pain 256 mN	11	10.9	t = 0.03	0.98
Precapsaicin Suprathreshold Mechanical Pain 512 mN	12.7	13	t = 0.07	0.94
Capsaicin Heat Pain Model Exposure Temperature	39°C	39°C	t = 0.00	1.00
Heat Allodynia Pain Ratings	60	53	t = 0.94	0.35
Postcapsaicin Spontaneous Pain Rating	15.6	11.9	t = 0.83	0.41
Postcapsaicin Suprathreshold Mechanical Pain Rating 64 mN	15.4	11	t = 0.96	0.35
Postcapsaicin Suprathreshold Mechanical Pain 128 mN	17.1	15.4	t = 0.35	0.72
Postcapsaicin Suprathreshold Mechanical Pain 256 mN	23.6	18.1	t = 0.96	0.34
Postcapsaicin Suprathreshold Mechanical Pain 512 mN	30.0	22.1	t = 1.25	0.22
Area of Secondary Mechanical Hyperalgesia	50.9	44.6	t = 0.55	0.58
Short Form McGill Pain Questionnaire-2 Sum	36.8	32.7	t = 0.48	0.63

Supplemental Table 3. Voxel table of contrast map of mechanical pain-related activation during the sham tDCS session where post-C-HP > pre-C-HP.

Brain Region	Side in Reference to Stimuli	Brodmann Area	Volume	Maximum Intensity	TLRC Coordinates
Right Posterior Cingulate	ipsilateral	BA30	4266	-5.5	(17, -59, 12)
Right Parahippocampal Gyrus	ipsilateral	BA36	1539	-5.7	(26, -32, -13)
Right Fusiform Gyrus	ipsilateral	BA37		-5	(44, -50, -13)
Left Anterior Cingulate	contralateral	BA32	1269	-4.4	(-8, 44, -7)
Left Middle Temporal Gyrus	contralateral	BA39	1080	-5	(-44, -68, 21)
Left Inferior Frontal Gyrus	contralateral	BA44	945	4.7	(-56, 17, 18)
	contralateral	BA44		4.5	(-50, 11, 12)
Left Precentral Gyrus	contralateral	BA44		4.3	(-50, 8, 12)
Left Posterior Cingulate	contralateral	BA29	918	-4.7	(-14, -50, 6)
Right Superior Frontal Gyrus	ipsilateral	BA10	918	-4.7	(2, 59, 12)
Right Middle Temporal Gyrus	ipsilateral	BA22	783	-4.5	(62, -32, 3)
Left Postcentral Gyrus	contralateral	BA2	756	4.6	(-53, -23, 33)
Right Insula	ipsilateral	BA41	567	-5.3	(41, -23, 12)
Right Insula	ipsilateral	BA13		-4.7	(35, -20, 24)
Right Cerebellar Declive	ipsilateral		378	4.4	(14, -71, -19)
Left Putamen	contralateral		351	4.4	(-26, 2, -4)
Right Medial Globus Pallidus	ipsilateral		189	-3.6	(11, 2, -4)
Right Thalamus (LP)	ipsilateral		189	-3.7	(17, -20, 15)
Ventral Pons	contralateral		162	-5.7	(-8, -20, -40)
Left Thalamus (Pul)	contralateral		162	-4.9	(-11, -23, 9)
Left Caudate	contralateral		108	-3.5	(-5, 2, 6)
Right Thalamus (MD)	ipsilateral		108	-3.2	(5, -20, 6)

Supplemental Table 4A. Voxel table of contrast map anodal compared to sham tDCS.

Brain Region	Side in Reference to Stimuli	Brodmann Area	Volume	Maximum Intensity	TLRC Coordinates
Left Medial Frontal Gyrus	contralateral	BA10	810	4.9	(-2, 53, 6)
Left Precentral Gyrus	contralateral	BA4	783	-4.4	(-56, -17, 36)
Right Pontine nuclei	Ipsilateral		108	3.4	(14, -26, -34)
Right Caudate	Ipsilateral		108	3.6	(8, 5, 6)

Supplemental Table 4B. Voxel table of contrast map cathodal compared to sham tDCS.

Brain Region	Side in Reference to Stimuli	Brodmann Area	Volume	Maximum Intensity	TLRC Coordinates
Right Medial Frontal Gyrus	Ipsilateral	BA6	1431	-6.6	(8, -14, 72)
Left Insula	Contralateral	BA13	702	-5.3	(-41, -8, 6)
Left Cingulate Gyrus	Contralateral	BA31	702	-4.9	(-8, -8, 45)
Rostral Medulla	Ipsilateral		324	-5.9	(2, -35, -40)
Rostral Pons/Midbrain/ Red Nucleus	Ipsilateral		324	-4.2	(8, -23, -16)
Right Medial Globus Pallidus	Ipsilateral		189	4.4	(20, -8, -7)
Right Thalamus	Ipsilateral		189	-4	(11, -14, 6)
Right Caudate	Ipsilateral		162	3.9	(11, 5, 6)

Supplemental Table 5A. Treatment main effect and treatment by state interaction of linear mixed model of BOLD response to pin prick including sensitization state, intervention type and probe force as fixed effect factors (without interactions involving force) and probe force and session order nested within subject as the random effect factors.

ROI	Treatment by State F-stat	Treatment by State p-value	Treatment F-stat	Treatment p-value
Anterior Midcingulate Cortex	1.23	0.27	0.82	0.37
Pregenual Anterior Cingulate Cortex	2.38	0.12	0.23	0.64
Periaqueductal Gray	0.62	0.43	0.022	0.88
Medial Prefrontal Cortex	9.66	0.0021	5.79	0.017
Left Somatomotor Cortex	8.49	0.0039	4.68	0.031

Supplemental Table 5B. Treatment main effect of linear mixed model of the change in BOLD response during the session (post-capsaicin/post-tDCS minus pre-capsaicin/pre-tDCS; change score analysis (CSA)) to pin prick including intervention type and probe force as fixed effect factors (without their interaction) and probe force and session order nested within subject as the random effect factors.

ROI	Treatment F-stat	Treatment p-value
Anterior Midcingulate Cortex	2.81	0.096
Pregenual Anterior Cingulate Cortex	0.96	0.33
Periaqueductal Gray	1.46	0.23
Medial Prefrontal Cortex	6.25	0.014
Left Somatomotor Cortex	6.79	0.010

Supplemental Table 5C. Treatment main effect of linear mixed model of the pretreatment BOLD response to pinprick including intervention type and probe force as fixed effect factors (without their interaction) and probe force and session order nested within subject as the random effect factors.

ROI	Treatment F-stat	Treatment p-value
Anterior Midcingulate Cortex	0.089	0.77
Pregenual Anterior Cingulate Cortex	2.23	0.14
Periaqueductal Gray	1.03	0.31
Medial Prefrontal Cortex	0.086	0.77
Left Somatosensory Cortex	0.34	0.56

Supplemental Table 6A. Voxel table of covariation map of pain intensity with BOLD response evoked by painful mechanical stimuli after sham tDCS.

Brain Region	Side in Reference to Stimuli	Brodmann Area	Volume	Maximum Intensity	TLRC Coordinates
Left Middle Temporal Gyrus	contralateral	BA19	1728	5.2	(-47, -62, 12)
Right Lingual Gyrus	ipsilateral	BA18	1674	5.1	(11, -77, 6)
Left Lingual Gyrus	contralateral	BA18	1242	4.6	(-8, -80, 3)
Right Posterior Cingulate	ipsilateral	BA30	999	4.3	(14, -53, 12)
Left Medial Frontal Gyrus	contralateral	BA6	999	6.6	(-5, -17, 66)
Right Insula	ipsilateral	BA13	837	4.3	(35, -23, 18)
Left Superior Temporal Gyrus	contralateral	BA22	675	4.5	(-50, 5, 6)
Left Cingulate Gyrus	contralateral	BA32	567	4.9	(-8, 17, 36)
Right Thalamus (MD)	ipsilateral		540	4.9	(8, -17, 6)
Right Thalamus (Pul)	ipsilateral		297	5	(2, -29, -1)
Left Thalamus (Pul)	contralateral		270	4.3	(-8, -23, 9)
Left Caudate	contralateral		135	4.9	(-8, 2, 6)

Supplemental Table 6B. Voxel table of covariation map of pain intensity with BOLD response evoked by painful mechanical stimuli after anodal tDCS.

Brain Region	Side in Reference to Stimuli	Brodmann Area	Volume	Maximum Intensity	TLRC Coordinates
Left Precentral Gyrus	contralateral	BA44	621	6.1	(-56, 8, 6)
Right Cerebellar Inferior Semilunar Lobule	ipsilateral		378	4.7	(20, -71, -40)
Left Thalamus	contralateral		135	4.3	(-20, -23, 9)

Supplemental Table 6C. Voxel table of covariation map of pain intensity with BOLD response evoked by painful mechanical stimuli after cathodal tDCS.

Brain Region	Side in Reference to Stimuli	Brodmann Area	Volume	Maximum Intensity	TLRC Coordinates
Left Medial Frontal Gyrus	contralateral	BA6	3078	5.2	(-2, -23, 54)
Left Paracentral Lobule	contralateral	BA5		5.2	(-2, -38, 60)
Left Postcentral Gyrus	contralateral	BA5		4.9	(-8, -41, 72)
Right Precentral Gyrus	ipsilateral	BA6	783	5	(56, -2, 27)
Right Paracentral Lobule	ipsilateral	BA4	567	4.2	(8, -35, 72)
Left Putamen	contralateral		270	4.5	(-23, 14, 3)
Right Putamen	ipsilateral		216	4.1	(26, 5, -4)
Right Lateral Globus Pallidus	ipsilateral		216	-4.1	(20, -2, 3)
Right Putamen	ipsilateral		216	4.4	(20, 8, 9)
